# Human-to-human closed-loop control based on brain-to-brain interface and muscle-to-muscle interface

**DOI:** 10.1038/s41598-017-10957-z

**Published:** 2017-09-08

**Authors:** M. Ebrahim M. Mashat, Guangye Li, Dingguo Zhang

**Affiliations:** Institute of Robotics, School of Mechanical Engineering, Shanghai Jiao Tong University, Shanghai, 200240 China

## Abstract

Novel communication techniques have always been fascinating for humankind. This pilot study presents an approach to human interaction by combining direct brain-to-brain interface (BBI) and muscle-to-muscle interface (MMI) in a closed-loop pattern. In this system, artificial paths (data flows) functionally connect natural paths (nerves). The intention from one subject (sender) is recognized using electroencephalography (EEG) based brain-computer interface (BCI), which is sent out to trigger transcranial magnetic stimulation (TMS) on the other subject (receiver) and induce hand motion; meanwhile TMS results in a significant change on the motor evoked potentials (MEP) recorded by electromyography (EMG) of the receiver’s arm, which triggers functional electrical stimulation (FES) applied to the sender’s arm and generates hand motion. Human-controlled loop and automatic control loop experiments were performed with 6 pairs of healthy subjects to evaluate the performance of the introduced mechanism. The results indicated that response accuracy during human-controlled experiments was 85% which demonstrates the feasibility of the proposed method. During the automatic control test, two subjects could accomplish repetitive and reciprocal hand motion control up to 85 times consecutively.

## Introduction

The conventional interactions between two humans or animals basically depend on vision, audition, voice, olfaction or touch. However, new technologies, such as brain-to-brain interface (BBI) and muscle-to-muscle interface (MMI), have been proposed based on unconventional approaches to explore the novel concept of interactive communication^1, 2^. BBI, which emerged as an extension of brain-computer interface (BCI), aims to transfer information between two individuals merely using their brains without any intentional physical motion. This technique was first tested on communication among two brains of a pair of functioning rats to jointly learn and move in synchrony. In this study M1 neural ensemble was used as a motor information elicitation source in the encoder rat and invasive intercortical microstimulation (ICMS) as corresponding command inducer in the decoder rat’s brain^3^. BBI was also used to successfully establish artificial information transfer pathway from a human to a rat using electroencephalography (EEG) and transcranial focused ultrasound (FUS) to control a simple motion of the rat’s tail using BBI^4^. In a later study, the first direct brain-to-brain interface between two humans was established, which investigated the feasibility of decoding a command from a sender’s brain by EEG and forcing a receiver to follow the command using transcranial magnetic stimulation (TMS)^1^. Analogously, internet-based human brain-to-brain communication also succeeded in implementing conscious word transmission, over a long distance, exploiting EEG and TMS by means of Bacon’s cipher^5^. BBI has inspired other interesting application, by adopting steady-state visual evoked potential (SSVEP)-based BCI for the human and applying invasive neural stimulation to a cyborg cockroach, the human could control the cockroach to walk along a trajectory^6^. Although some achievements have been accomplished through establishing functional BBIs, all the previous work just focused on single-way communication and realized open-loop control between two subjects, i.e. only the sender can control the receiver.

**Figure 1:**
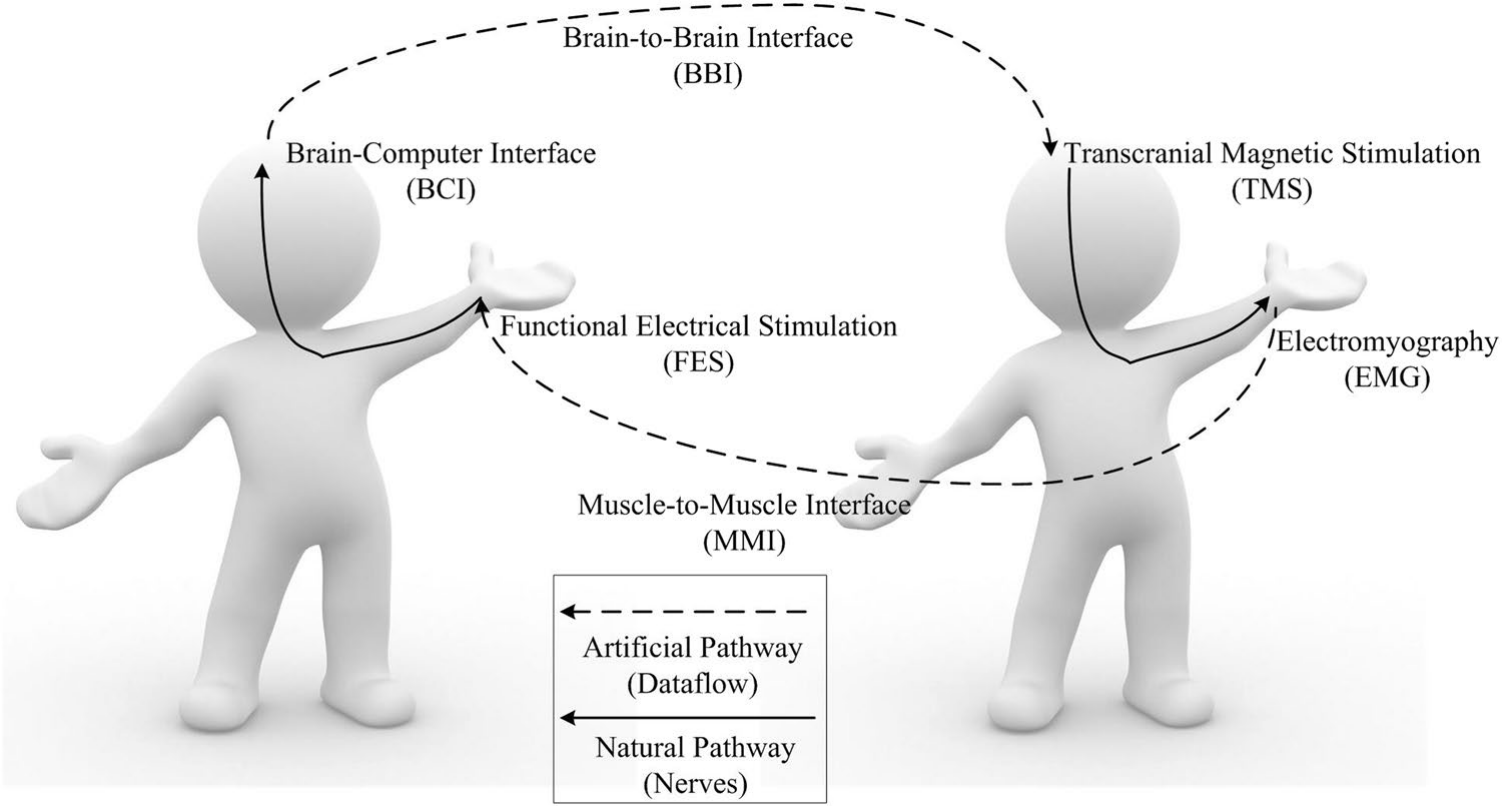
Original idea and basic terms/ acronyms of the human-to-human closed-loop control based on brain-to-brain interface and muscle-to-muscle interface.

In addition, an engrossing artificial communication technique is used for building an information path between two muscles, we call it muscle-to-muscle interface (MMI) herein. In the rehabilitation field, MMI is generally introduced by using electromyography (EMG) for functional electrical stimulation (FES) control^7^. In previous studies, EMG-controlled FES was mostly applied to muscles of one and the same subject for rehabilitation^8^. Recently, EMG-controlled FES was used in a master-slave paradigm between two persons, indicating as well that MMI can be a feasible information transfer approach for human-to-human control^2^. Despite the fact that BBI and MMI technologies have separately substantiated as novel ways to establish artificial communication between two functioning organisms, the result when using them in a unified mechanism remains obscure. This work aims to develop closed-loop control between two persons based on BBI and MMI as shown in Fig. 1. We expected to build two artificial pathways that functionally connect two natural neural pathways as a closed information loop. We adopted EEG-based BCI and TMS to construct one artificial pathway (i.e. BBI), and EMG-triggered FES to form the other one (i.e. MMI). FES can evoke EEG through afferent nerves in one person, and TMS can induce EMG through efferent nerves in the other person. The realization, as well as the performance of the current system, is presented in this paper.

## Results

### Latency and repeatability

**Figure 2:**
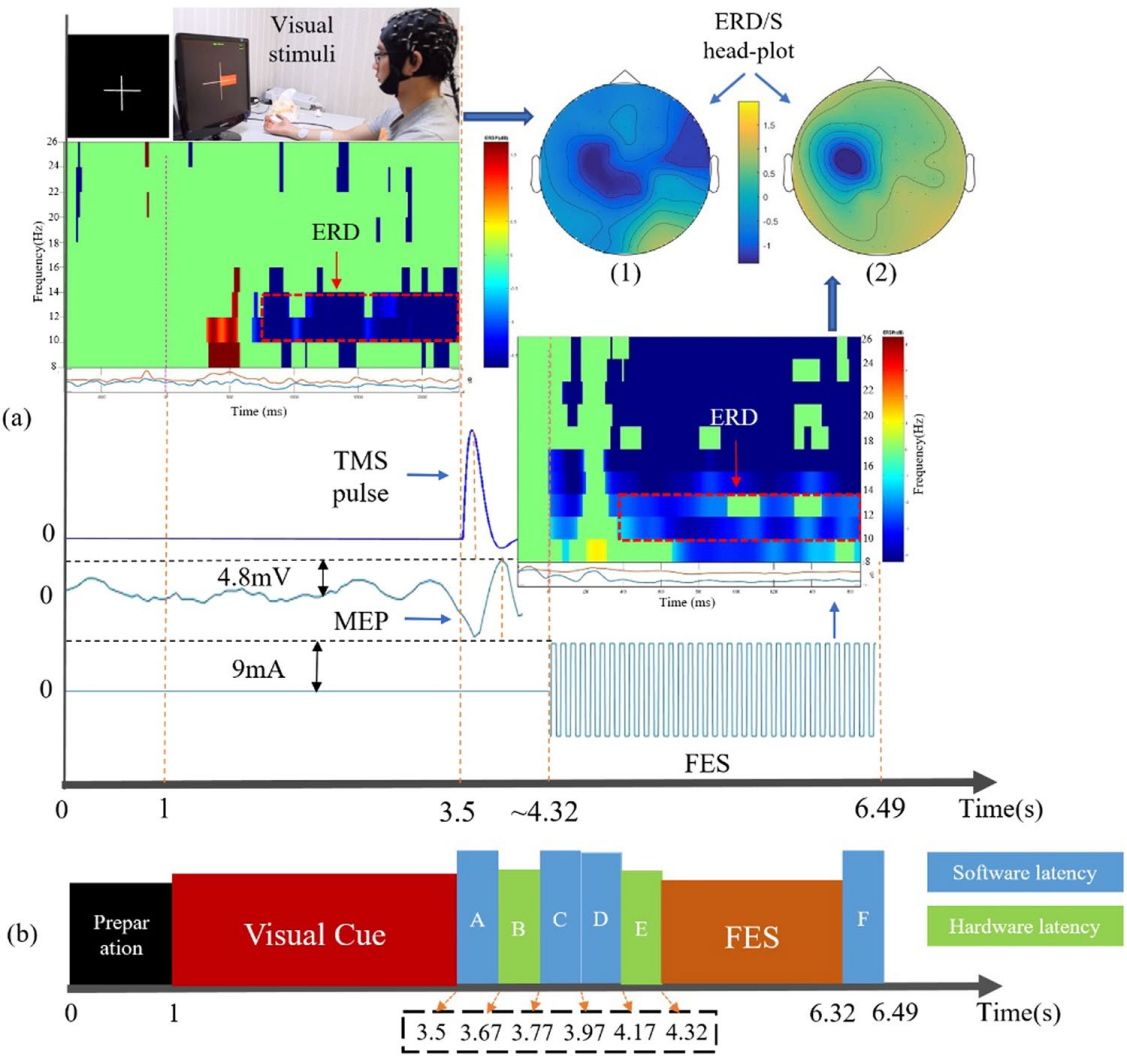
Step-by-step data flow and latency in a representative trial. (**a**) different steps of the experiment in detail and signals driving different devices are shown. Brain activations are illustrated by spectral power attenuation (ERD), in form of head plots and time-frequency maps, showing the location and frequency bands of cortical activations, during right hand MI and FES-evoked BCI. TMS pulse shape, induced MEP magnitude, and FES stimulation pulse are illustrated. (**b**) latencies for each step including preparation and visual cue appearance, A and F are EEG data classification latencies, B is trigger transfer delay via network, C is EMG classification latency, and D and E are delays for FES driver software to receive trigger and hardware to start stimulation, respectively. Physiological delays (i.e. delay of neural pathway from brain to hand muscle) were not detectable. Blue values are software related latencies. Green values are hardware-related latencies.

The representative results are shown in Fig. 2(a). Brain activations are reflected by event-related de-synchronization (ERD)^9^, which is a normalized power attenuation in a specific frequency band (upper alpha rhythm 10–13(Hz)) with respect to a baseline time window (0.8–0.1(s) prior to the placement of cue). Motor cortical activations in form of 2-D head plots are illustrated to vividly describe the underlying mechanism of the brain and how it interacted with the mechanism. Head plots - drawn using FieldTrip, an open source MATLAB package - indicate activation spots over the brain according to electrode positioning. The majority of activations for both head plots are in the vicinity of C3 electrode over the contralateral hemispheric motor area of the right hand side. A time-frequency map of channel C3 is shown to illustrate a point to point brain activation in the contralateral hemisphere during MI task. Time-frequency plots are employed for this purpose which are plotted using EEGlab (open source MATLAB toolbox). Furthermore, sequential steps with their corresponding time delay are illustrated in Fig. 2(b). Accordingly, each loop takes 6.49 seconds in total to perform all steps (excluding rest time). Although BCI classification part had a 0.17 s delay, trigger circuitries did not introduce any detectable time delay while trigger signal transmission through the Internet showed 0.1 s latency. However, a large portion of the delay (4.5 s) corresponds to MI-BCI task and FES-evoked BCI. Despite the complexity of the mechanism design, hardware- (i.e. connections, network, circuits, processing times, etc.) and software-related (i.e. signal processing algorithm, device drivers, system software, etc.), latencies were negligible (merely 0.99 s). This fast response time corresponds to the short delay of BCI classification, EMG-triggered FES detection, and network data transfers which were approximately 0.17 s, 0.35 s, and 0.1 s, respectively. The interface between BBI and MMI was established using natural neural network connection from contralateral hemispheric motor area to index finger muscle with a trifling delay.

Additionally, automatic control loop applied to a representative pair (pair 6), demonstrated the two subjects successfully completed 85 loops without any break upon just one time motor imagery initiated by the subject in side A, which conclusively confirms the repeatability of the mechanism.

### System performance

**Figure 3:**
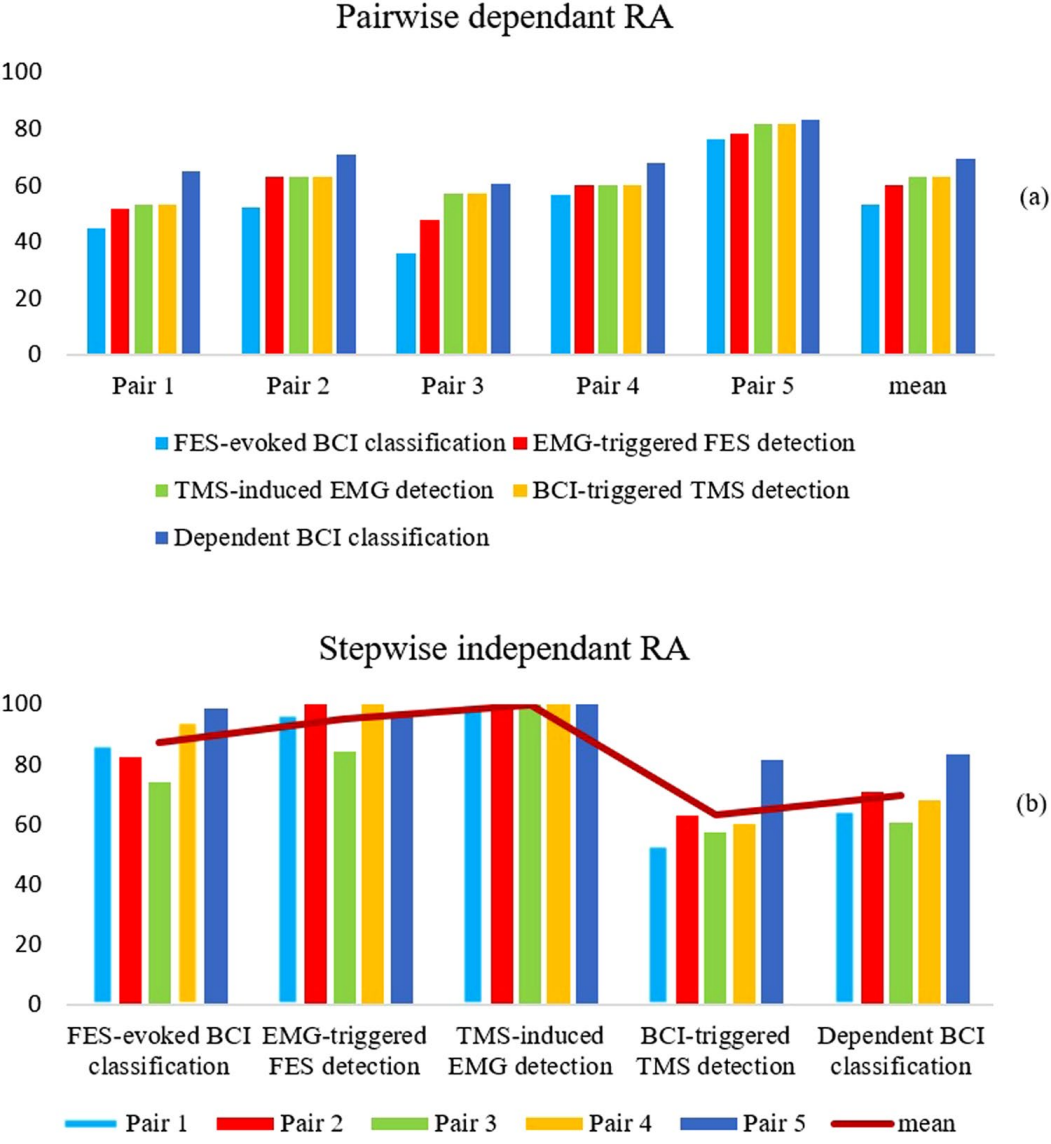
Response accuracy corresponding to each pair illustrated in form of accuracies. All accuracies are the average values through 120 trials for each pair. (**a**) Pairwise accuracies are calculated with respect to the prior step. In this experiment, the performance of each step is absolutely dependent on the previous one. (**b**) Step-wise accuracies for each pair are calculated without respect to prior step to illustrate the difference between MMI and BBI part.

For right-hand true detection, the accuracy is calculated with the number of successful detections to the number of visual cues instructing the same hand motion imagination. Each session consists of 30 trials, among which half trials visual cue is on the right and half on the left. Figure 3(a) illustrates overall independent response accuracy (RA) for 4 sessions (120 trials) of the experiment for each pair. Each loop is categorized into four independent steps and a dependent step of human-controlled paradigm. Accuracy for each step is calculated by:

1 $$RA={N}_{TT}/{N}_{RT}\ast 100$$

where the number of trials with a true response for a specific step is *N*_*TT*_ and the number of related trials in each step is *N*_*RT*_. Here dependent RA corresponds to responses recorded for each step with respect to the prior step, for instance, the accuracy for BCI-triggered TMS detection is calculated with respect to the number of true right-hand motor imagery classification, therefore, we call aforementioned accuracies dependent accuracy. Meanwhile, independent RA is defined to show the efficiency of each individual step without any respect to its previous step. Since the number of visual cues on the right and left in each session is equal, the number of related trials for dependent BCI is 30 and for the rest of steps is 15. Dependent RA for each pair is shown in Fig. 3(a). As depicted from the figure, pair 5 has the best mean RA (80%) while the value of pair 3 is the worst (53%). Meanwhile, the highest RA reaches as high as 93%, occurred in the 3rd session of pair 5. Step-wise accuracies for each pair (Fig. 3(b)) are obtained, according to the results, MMI showed superior outcome to BBI for all pairs.

The overall RA is 69.7 ± 8.55% and 87.37 ± 9.7% for BBI and MMI segments, respectively, which suggests muscle to muscle interface with the current mechanism is more reliable and accurate than the current BBI mechanism. The total response rate of the mechanism over 20 session shows 85.7 ± 12.13% accuracy. Notably, the accuracy of TMS-induced EMG detection is always 100%, owing to the fact that whenever right-hand motor imagery detection is true, a trigger is sent to TMS. The same situation is valid for EMG-triggered FES detections. The difference between pairs mostly depends on hand motor imagery and passive movement induced by FES classification accuracy. The rest of the system is properly operating which is in the prospect of a reliable BBI and MMI system. Overall RA of FES-induced BCI classification in the offline analysis have the average of 84.5% while for motor imagery classification is 63% totally (for stop and start commands). Conclusively, the overall FES-induced BCI classification accuracy was higher than motor imagery.

### Analysis of variance

The Independency Ratio (IR) is defined between 0 and 1, as an indication measure for each step individually, regardless of the performance of prior step. IR can be calculated with the following equation:

2 $${IR}={accuracy}\,{of}\,{step}({n})/{accuracy}\,{of}\,{step}(n-1)$$

Considering the hierarchy of steps, efficiency of each step independently without the influence of previous one needs to be calculated separately. To address this, analysis of variance (ANOVA) of independency ratio over different steps using results from 20 sessions of the experiment is conducted. Stepwise ANOVA yields a p-value of 0.00025 which is dramatically smaller than the level of significance (alpha = 0.05). Thus, the null hypothesis, namely “the efficiency of each step is based on chance”, is rejected and results are statistically significant (F-value 5.908). The first step was excluded from IR assessment, since its independent accuracy is unconditionally pertinent to subject-related BCI performance, rather than system performance. Therefore, calculations started from second step, which illustrated *IR* = 0.87 ± 0.12 with 0.015 variance, while the third step perfectly completed without any misses (IR = 1). Meanwhile, fourth and fifth steps, demonstrated *IR* = 0.97 ± 0.06 with 0.003 variance and *IR *= 0.878 ± 0.13 with 0.01 variance, respectively. Hence, individual steps functioned efficiently.

### Receiver operating characteristics

**Figure 4:**
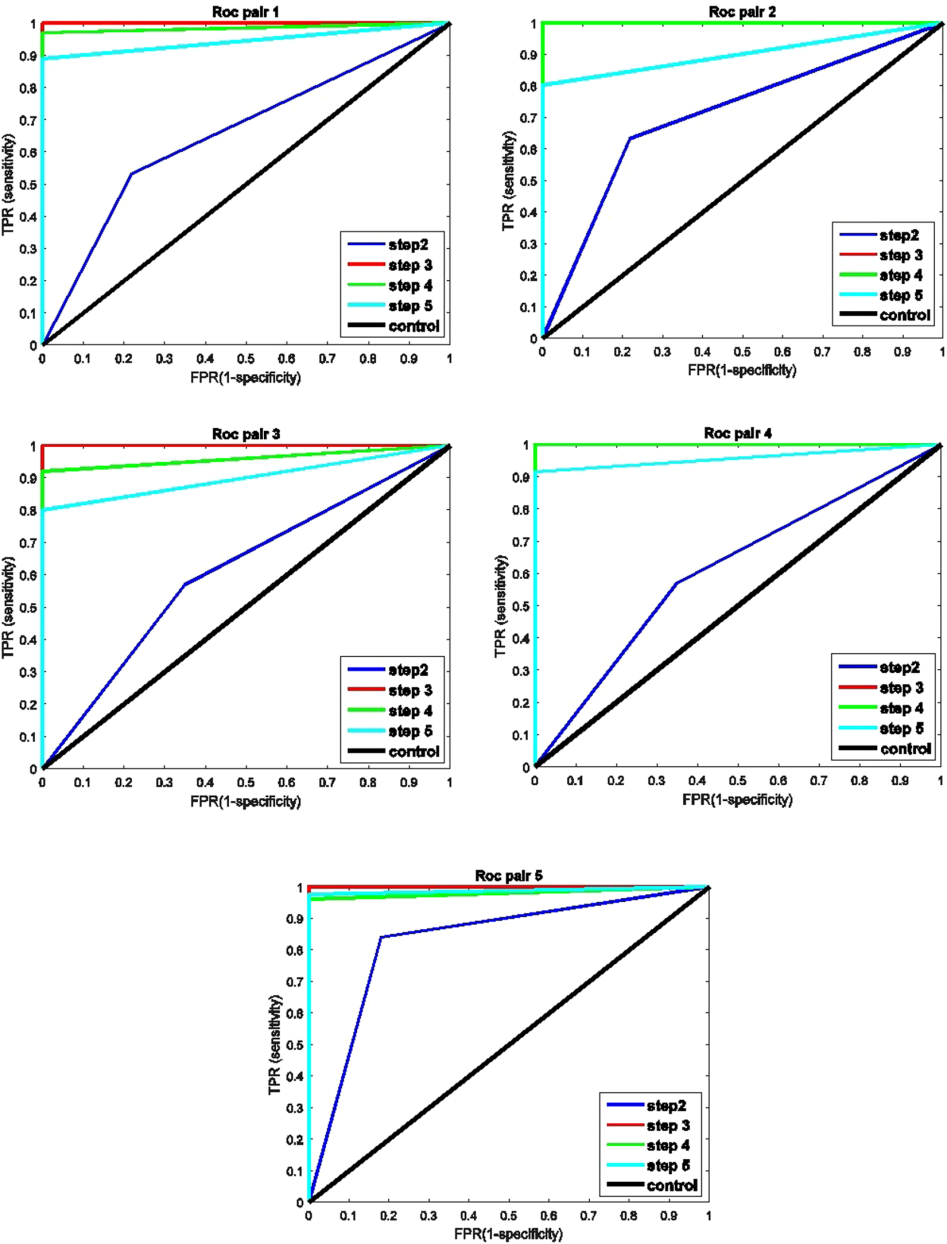
ROC curves related to step 2 (BCI-triggered TMS detection), step 3 (EMG-triggered FES detection), and step 5 (FES-evoked BCI) plotted for each pair according to their TPR and FPR values. Blue, red, and green lines are test experiment results of steps 1 to 3, respectively, while the black line is control result.

To better quantify the system performance, the receiver operating characteristics (ROC) curve is drawn for each pair in Fig. 4. The efficacy of each step of this experiment is depicted using True Positive Rate (TPR) and False Positive Rate (FPR)^10^. Thus, some measures are defined. True Positive (TP) is true command sent to the receiver and true movement detected; False negative (FN) is true command sent to the receiver but false movement detected; False Positive (FP) is false command sent to the receiver but true movement detected; True negative (TN) is false command sent to the receiver and false movement detected. It is noteworthy that the mentioned values have been calculated solely regardless of prior steps. The accuracy of the test depends on how well the test separates the group being tested into detected and not detected trials. Accuracy is measured by calculating the area under the ROC curve (AUC). The closeness of the curve to the top-left corner of the ROC chart depicts superior performance. This figure gives a good comparison between control session and experimental sessions, with step-wise contrast. The lowest efficacy in all stages of the experiment is 0.6 of full AUC corresponding to step 2 of pair 3, which is higher than the area of control experiment (0.5). Although the largest area under the curve is 1, the best MMI + BBI efficacy is related to pair 5 with overall 0.94 ± 0.08 of AUC. Step-wise analysis illustrates that step 3 with 1.0, step 4 with 0.977 ± 0.028, step 5 with 0.924 ± 0.044, and step 2 with 0.68 ± 0.09 of AUC present best to worst performances, respectively. Conclusively, test experiments for all pairs had a larger AUC than control ones, which is expected from a working system.

### Mutual information transfer

Mutual information^11^, as an efficiency indicator of information transferred between two subjects, is carried out to evaluate the performance of the current system. The amount of mutual information transferred is calculated by multiplying response vector I(A, B) with the number of related trials. Here, the whole system is disassembled into two parts. In the first part, we obtained the mutual information transferred from the subject’s brain (side A) to the other subject’s muscle (side B), as illustrated in Table 1. The results demonstrate that the average information transferred via brain to brain to muscle varies between 18.82 ± 7.99 bits for pair 3 and 26.62 ± 4.432 bits for pair 5. Moreover, for the second part, mutual information transferred from the subject’s muscle (side B) back to the subject’s brain (side A) are determined and shown in Table 2. According to this table, information transferred in the remaining chain via brain to brain to muscle vary from 9.22 ± 1.27 bits for pair 1 to 10.82 ± 2.73 bits for pair 5. Even though there is common inter-subject variability for BCI dependent step, remaining steps are perceived to work reliably. In previous results, it is indicated that MMI shows a better performance than BBI, however, less mutual information is transferred through MMI herein. This occurrence is basically due to sequential nature of the mechanism which allows only those trials with a prosperous BBI to reach MMI section, which means half of the trials are related to the second part. Thus, with respect to the linear correlation between the transferable amount of mutual information and number of trials, less information can be transferred in MMI to brain section. Accordingly, there is no contradiction between results.

**Table 1:** First number is response vector value for a whole session I(A, B), numbers in parenthesis indicates *mean* ± *std* mutual information (in bits) transferred between subject A’s brain and subject B’s hand (BBI to muscle)(first column) and between hand of subject in side B and brain of subject in side A (MMI to brain) (second column).

Pair	BBI to Muscle	MMI to Brain
Pair 1	0.76 (23 ± 3.77)	0.61 (9.22 ± 1.27)
Pair 2	0.89 (26.7 ± 1.4)	0.67 (10.1 ± 3.53)
Pair 3	0.62 (18.9 ± 7.9)	0.62 (9.45 ± 4.1)
Pair 4	0.83 (25 ± 4.02)	0.67 (10.15 ± 4.69)
Pair 5	0.88 (26.6 ± 4.43)	0.71 (10.8 ± 2.73)

The amount of mutual information transferred is calculated by multiplying response vector with the number of related trials which is 30 and 15, for BBI to muscle and MMI to the brain, respectively.

**Table 2:** Information about participants in the experiment.

Pair	Role in BBI	Role in MMI	Age (Y)	Gender (M/F)
Pair 1	sender	receiver	24	M
Pair 1	receiver	sender	25	M
Pair 2	receiver	sender	26	M
Pair 2	receiver	sender	25	M
Pair 3	sender	receiver	21	M
Pair 3	receiver	sender	21	M
Pair 4	sender	receiver	20	M
Pair 4	receiver	sender	21	M
Pair 5	sender	receiver	21	M
Pair 5	receiver	sender	20	M
Pair 6	sender	receiver	22	M
Pair 6	receiver	sender	21	M

## Discussion

A novel and distinguishing communication method based on BBI and MMI is demonstrated herein. Our current results indicate the possibility of functionally combining natural and artificial neural pathways to establish a novel communication path. It has two sections, first, from one person’s brain towards another person’s brain and muscle, second, from one person’s brain to their own hand. This is accomplished by merely using noninvasive technologies with short latency and reliably working mechanism. In other words, the goal was to establish a composite neural pathway using human natural and BCI-based artificial neural pathway to transmit commands from a sender’s brain to his muscle. To this end, cortical activations decoded from EEG and muscle movements detected from EMG signal were used as inputs, while FES and TMS were used as outputs of the artificial neural system. Experimental outcomes showed up to 85% of mean system RA achieved and totally 37.44 ± 2.5 bits of mutual information transferred. Furthermore, mean IR of steps 2 to 5 is 0.96 ± 0.08 and 0.99 s delay, explicitly reveals the system reliability and relatively fast response time of different steps of the mechanism. The proposed system may provide interesting entertainment between two individuals. To our knowledge, intending to make a move and use natural neural pathway possessed by another person to induce movement command in the first person’s muscle is not investigated in any previous research work. The novel paradigm introduced in this paper proposes a new human-to-human communication way which engages human brain and muscles. A promising capability of this mechanism is the interaction between two subjects from any distance, using the Internet as a part of the artificial neural pathway. In this way, simultaneous learning of two or more persons who are far apart is conceivable. Although analogous works focused on BBI or MMI alone have been accomplished, the full interaction between two subjects has never been addressed in any previous works in the field. Our results suggest that the proposed procedure of the mechanism is performing satisfactorily. Thus, the current design of combining artificial and neural pathways to unconventionally communicate two persons is adequately sufficient and can be subject to find operational applications.

A substantial objective of the BCI-based research is literally for rehabilitation. In addition to its entertainment application, currently proposed system can be used fundamentally for rehabilitation. Four well-known technologies used in this system, BCI, TMS, EMG, and FES are popular rehabilitation technologies. In former studies, the four employed technologies are widely used independently. Some researchers have attempted to combine two technologies among them for rehabilitation. For instance, BCI have been combined with FES^12^ and TMS^13^. Additionally, MI-based BCI has been proven to effectively improve motor recovery^14^. BCI can decode the motion intention and control related devices, in this case, the patients are in a rather active role in the rehabilitation process. It might potentially enhance the rehabilitation of stroke-related motor impairments by promoting neuroplasticity and altering motor cortical areas. Therefore, rehabilitation is likely to be remarkably improved^15^. Our proposed method initially combined all the four methods in a closed-loop system. The two subjects in side A and side B can exchange and experience all the four kinds of rehabilitation technologies. Majority of current rehabilitation procedures are performed solely on a single patient, but our research can engage two patients to realize co-rehabilitation as double players in a game. The co-rehabilitation could be designed as a competitive way, which means both patients in side A and side B are able to start or stop the rehabilitation process. They can compare “who win or who lose”. They can also exchange their roles in side A and side B to play again. They may have strong desire to win, so the rehabilitation would be performed more enthusiastically. In such a way, the co-rehabilitation may become more attractive and interesting for the patients. In this work, a combination of BBI and MMI is accomplished to illustrate the possibility and applicability of this kind of rehabilitation, which is based on the reciprocal human to human interaction channel.

According to results, FES-evoked BCI demonstrated higher cortical activity within the demanded frequency bands (i.e. alpha and beta rhythm) compared to MI-based BCI as indicated by ERD plots depicted in Fig. 2(a) and FES-evoked BCI classification accuracy. This phenomenon might rely on the effect of FES-generated sensory feedback, which activates cerebellum and increases its interaction with cortex. Electrical stimulation of motor nerve fibers may generate both orthodromic and antidromic impulse. An impulse can cause depolarization of horn cells which leads to conductivity increase between pyramidal tract axons and anterior horn cells. Therefore, FES can induce changes in the segmental level even in people with lesioned limb and capable of activating anterior horn cell repeatedly which leads to enhancement in the corticospinal excitability compared to MI alone^16^.

The present work is a pilot study which has some limitations and there is a vast room for future development. Although noninvasive signal acquisition and stimulation methods employed in this paper are easy to implement and portable, their inherent shortcomings restrict further development of the mechanism. For instance, assessment of different natural neural pathway (i.e. lower limb motor nerves, etc.) effect is barely practicable. In addition, according to network based brain activations, for a perfect BBI all activated cortical spots in the brain of the subject in side A should be stimulated in the brain of subject in side B with corresponding latencies. However, it is impractical to stimulate different spots using a single TMS coil with low latency or using several TMS coils for neighboring spots. This is due to the size of the coil which prevents the reachability to certain regions that are spatially close to each other. Therefore, merely the physical response of the motor unit was used to determine which brain area to be excited. Moreover, only alpha and beta frequency bands are used to determine brain activations. This is mostly based on low signal to noise ratio of EEG signal which limits usability of features in other frequency bands (i.e. gamma band). Hence, noninvasive methods are replaceable with more accurate invasive technologies, i.e. Electrocorticography (ECoG), to accomplish a real task consisting of complex movements. Furthermore, implementation of the mechanism is technically complicated, this may pragmatically cause technical deficits while putting it into real-world application. Furthermore, simultaneous FES stimulation and MI suggested for maximizing short term neuroplastic effects^17^, while in our study they have been performed separately, which may be subject to modification in future rehabilitation practices.

Current software and hardware infrastructure is capable of being adopted for more than two healthy or disabled subjects to have interaction (e.g. social communication) with each other. Therefore, co-rehabilitation between two or more paralyzed patients simultaneously using the proposed mechanism can be further investigated. Besides, the response rate of the mechanism was directly interdependent to BCI performance. Thus, advanced sensorimotor pattern extraction algorithms to enhance the performance of the system and fundamental research on the exploration of the brain network that will provide more and effective control modes for BCI, are still in high demand. The current system design is one out of many possibilities. For instance, a possible substitution for BCI part is online ERD calculation. In this case, instead of using left or right hand EEG signal classification, attenuation or increase of ERD value in electrodes neighboring motor area can be used as BCI output and generate a trigger for TMS. Accordingly, TMS stimulation intensity could linearly be adapted with ERD value, meaning higher ERD value cause higher TMS stimulation intensity and vice versa. As a result, the amplitude of detected MEP will change linearly with TMS-induced movement, and accordingly, the FES stimulation may be modified and applies higher or lower stimulation intensity regarding MEP amplitude. Under this circumstance, both subjects will not only stop or start the mechanism but also control the range of movements which enhance the interaction level between two subjects.

## Methods

### Overview

**Figure 5:**
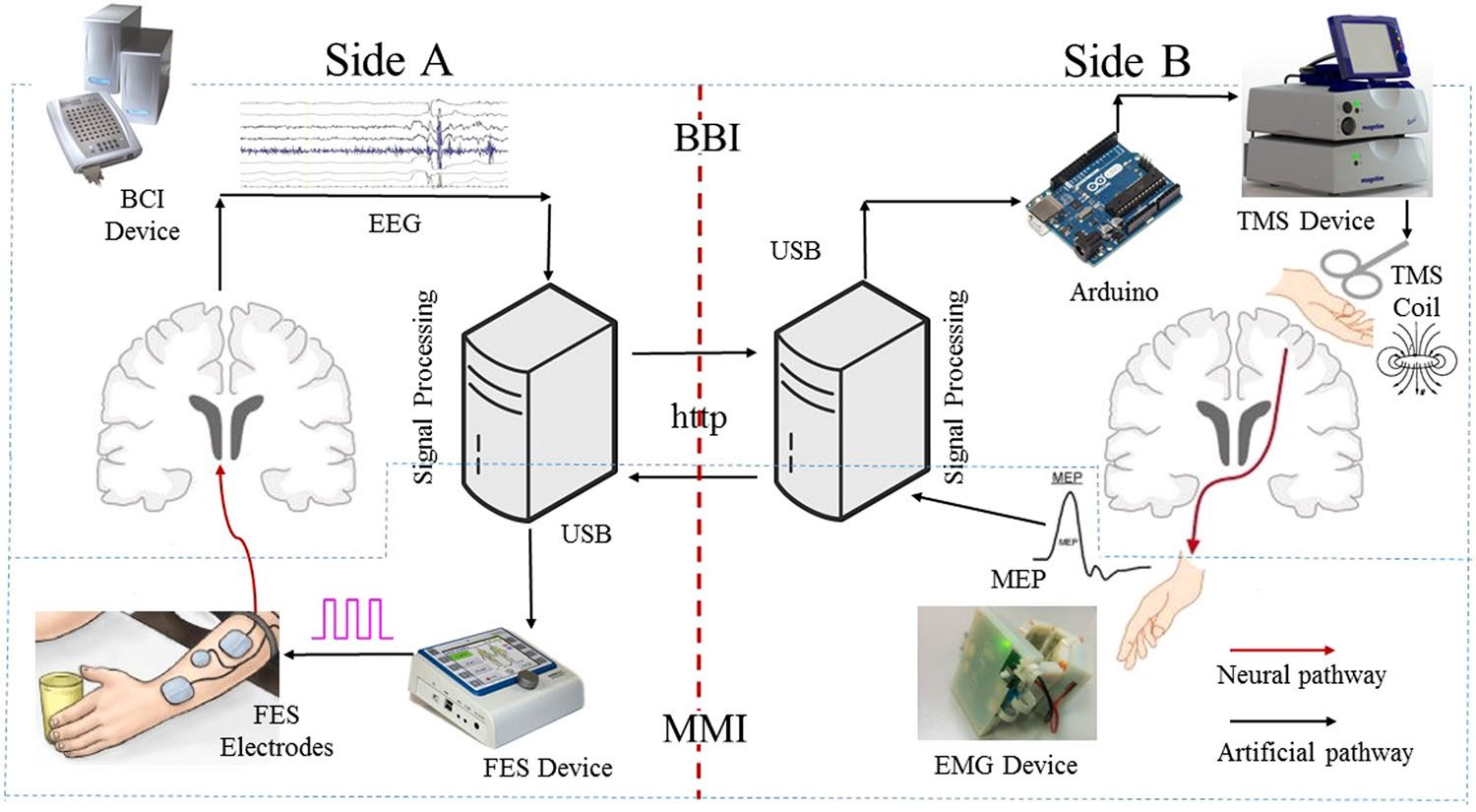
System implementation. Different steps of the mechanism, employed devices, connections and data flow direction is illustrated.

The implementation framework of BBI and MMI for human-to-human closed-loop control is shown in Fig. 5. In side A, we used a commercial device (SynAmp, Neuroscan, USA) for EEG measurement and a commercial device (REHASTIM 2, Hasomed, Germany) for FES. In side B, we used a commercial device (*Rapid* ^2^, Magstim, UK) for TMS, and a custom-made device (iMYO, SJTU, China) manufactured in biomechatronics and bio-robotics laboratory for EMG detection.

The proposed method consists of five main steps. First, the subject in side A uses motor imagery (MI, right or left hand motion) to start or stop the control (the first step: MI-based BCI classification). Afterwards, in the case that a required classification result is generated (i.e. right hand MI), a square pulse (5 V, 1000 ms) will be triggered as a handler of computer-to-brain interface (CBI) from an open-source electronic I/O board (Arduino, Sparkfun Electronics, Boulder, CO), subsequently initiates the operation of TMS device to non-invasively stimulate primary motor cortex of a subject in side B (the second step: BCI-triggered TMS). In the meantime, raw EMG data are collected and transmitted via Bluetooth 4.0 USB module to a desktop PC in side B, where runs a designated software (C#, Microsoft visual studio, USA) for EMG analysis, as a result of TMS, hand motion of the subject in side B may be elicited and in consequence will carry out a significant change in motor evoked potentials (MEP) compared to a threshold (the third step: TMS-induced EMG detection). MEP detection of hand movement will result in generation of a trigger signal and the signal will be sent to PC in side A via Internet connection (HTTP protocol), hence starting muscle stimulation on the subject in side A using a self-designated software coupled with FES device through USB connection (the fourth step: EMG-triggered FES detection). Finally, FES-generated hand motion may evoke changes on EEG signal which are detected via BCI system (the fifth step: FES-evoked BCI).

### Participants

This study was approved by local ethics committee of Shanghai Jiao Tong University. All experiments were performed in accordance with relevant guidelines and regulations. Twelve healthy subjects (age 23 ± 3 years old, all males), deprived of any disclosed background of central or peripheral nervous disease, voluntarily participated in this study (Table 2). All participants were naive to BCI and TMS operations. They were fully informed about the entire experimental procedure, the potential risks/benefits, and possibility of publishing their identifying information/images and thereupon given the written consents before the experiments. During the experiment, each subject in a pair played two roles, sender and receiver. Since the architecture of closed-loop system required the sender who sends command for BBI chain was also a receiver of MMI chain, and on the contrary, the receiver of BBI was also the sender of MMI chain.

### Brain-Computer Interface

BCI experiments were performed in an electromagnetic shielding chamber, which is designed for noise-sensitive experiments like EEG recording. The subject in side A wore an easy-cap of 64-channel Ag–AgCl electrodes (1 cm in diameter), with a right ear reference positioned on temporal bone and a ground electrode placed on the forehead next to front parietal. The electrodes were placed according to the international 10–20 system. All channels in the vicinity of the motor region on both hemispheres were used (C4, C2, CP4, C6, FC4, CZ, C1, C3, FC3, C5, and CP3). All impedances were kept below 5 *k*Ω throughout the experimental session. The signals were digitized at 250 Hz using a bio-signal amplifier (SynAmp, NeuroScan Inc, USA). The subject assigned to side A as a sender for BBI was seated in a comfortable chair and asked to put both the hands on the table in front of a PC display. They were instructed to do left or right-hand motor imagery (wrist flexion imagination) successively according to placement of a red rectangle cue as visual stimuli on the screen. Acquired raw EEG data were filtered using a 2nd-order infinite impulse response (IRR) Butterworth band-pass filter to the target frequency band (8–26 Hz) which covered the entire alpha and beta band as well as mu rhythm. Afterward, EEG features were extracted using common spatial patterns (CSP) method^18^. Extracted features were classified by the fisher’s Linear Discriminant Analysis (LDA) method^19^. Aforementioned algorithms are explained in details (Appendix A). A self-developed computer software in visual studio C++ (Microsoft Visual Studio, USA) was programmed to collect EEG data and sent it to a corresponding MATLAB code module (Mathworks, Natick, MA) simultaneously to automatically perform classification. The classified result was compared with the placement of visual cue, and the number of correctly classified trials was returned and shown on the screen.

### Transcranial Magnetic Stimulation

**Figure 6:**
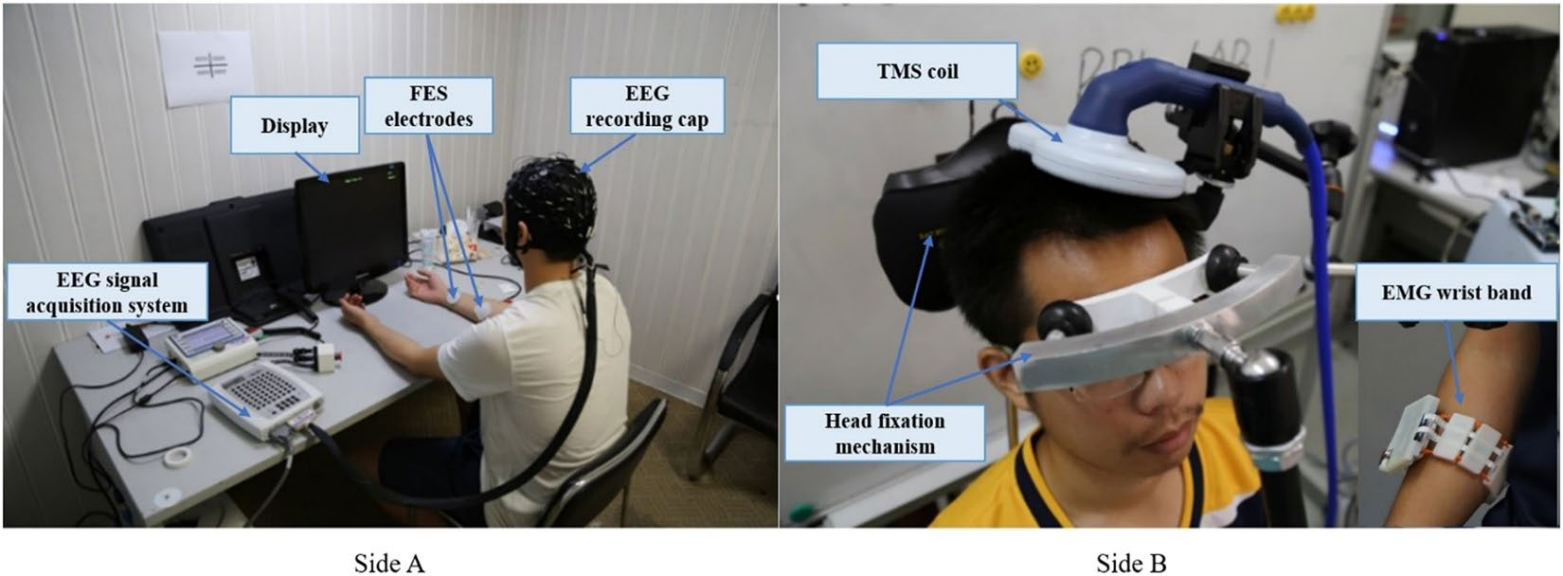
Illustration of experimental condition. The two subjects sat in two separate rooms to avoid any visual or auditory interactions. Side A: EEG equipment and FES device. Side B: Custom-designed head for TMS stimulation (the white part is soft and bouncy foam which is designed to reduce annoying tension on participant’s forehead). EMG wristband attached on the forearm shown on down-right.

TMS and repetitive TMS (rTMS) are non-invasive methods used to induce excitability changes in the motor cortex via a wire coil generating a magnetic field that passes through the scalp^20^. TMS is a technique that can non-invasively excite a specific population of cortical neurons with a spatial resolution of millimeters and a temporal resolution of microseconds^21^. Prior to performing the stimulation, an evaluation procedure was defined to optimally recognize the position of hand motor area on the contralateral hemisphere, M1 area on the scalp, which took approximately 10 minutes. Two participants were discarded from formal experiments and further analysis due to their unrecognizable hand motion response. Along the evaluation session, the subject in side B was accommodated on a comfortable chair specifically designed for fixating participant’s head handled by a mechanical mechanism. Head fixation was accomplished using a header stand and a forehead fasten part which can be adjusted according to each subject’s height and head size as shown in Fig. 6 (side B). TMS coil was centered tangentially over M1 hand motor area and fixed on the exact position and orientation discovered for each participant. The exact position (hot spot) for TMS over motor cortex, which connects to flexor carpi ulnaris muscle, was found manually by trial-and-error searching. Each single pulse was delivered by a double 70-mm coil (*Rapid2*, Magstim, UK). The stimulation intensity for each subject is expressed as a percentage of the maximum stimulation output (magnetic field strength 2.2 (Tesla) for double 70-mm coil), which were selected manually as follows regarding to each subject’s response to TMS stimulation: pair 1 (73%), Pair 2 (80%), pair 3 (75%), pair 4 (85%), pair 5 (75%), pair 6 (82%).

### Electromyography

A custom-made surface EMG sensor (iMYO, SJTU) consisting of 8 bipolar EMG channels (sampling rate 1000 Hz), designated on a soft wearable wristband as shown in Fig. 5, was used for measuring TMS-induced MEP fluctuations. Raw MEP data over flexor carpi ulnaris muscle were acquired from EMG sensor, amplified and transmitted to side B’s PC using Bluetooth communication system for further analysis. Feature extraction and classification were performed by a self-developed C# software (Microsoft visual studio). Acquired motion information was compared to a threshold value in real-time, defined as an average of MEP amplitude over 20 evaluation trials. Since insignificant changes in TMS-induced MEP were observed, the minor threshold value was picked (4–6 mV with inter-subject variability). The threshold value was optimally picked prior to the experimental session for each specific subject. The software would automatically figure out a proper motion was made in the subject’s hand, when the amplitude of four out of eight channels was evidenced to meet the threshold value and a trigger signal was sent to side A through an Internet connection (HTTP protocol).

### Functional Electrical Stimulation

FES, as a well-known noninvasive muscle stimulation method, is used in this experimental design. Rehabilitation and therapeutic effects of FES on gait, motor recovery, and motor cortex activity are widely studied in the literature^22^. There are also numerous interesting and unexplored applications of this method for moving a body joint spontaneously by stimulating skeletal muscles for healthy persons. In side A, FES was used to generate right-hand movement (wrist flexion), which was controlled by C++ program in PC via USB port. A pair of FES electrodes with approximate 25*cm* ^2^ was attached to the subject’s forearm skin over flexor carpi ulnaris muscle, and the stimulator delivers the desired electrical stimulation pulses to the electrodes through analog connections. The subject in side A was expected to perform the hand motion when the corresponding program received the trigger from side B. In each trial, as soon as a trigger was sent to TMS from side A, the software serving for FES looked for a trigger from side B. Once the intended trigger was received from EMG command, FES started the stimulation for a preset period of 2 seconds (biphasic square signal with 9 mA amplitude, 250 *μs* pulse width, and 30 Hz frequency).

### Experimental Protocol

#### Human-Controlled Loop

**Figure 7:**
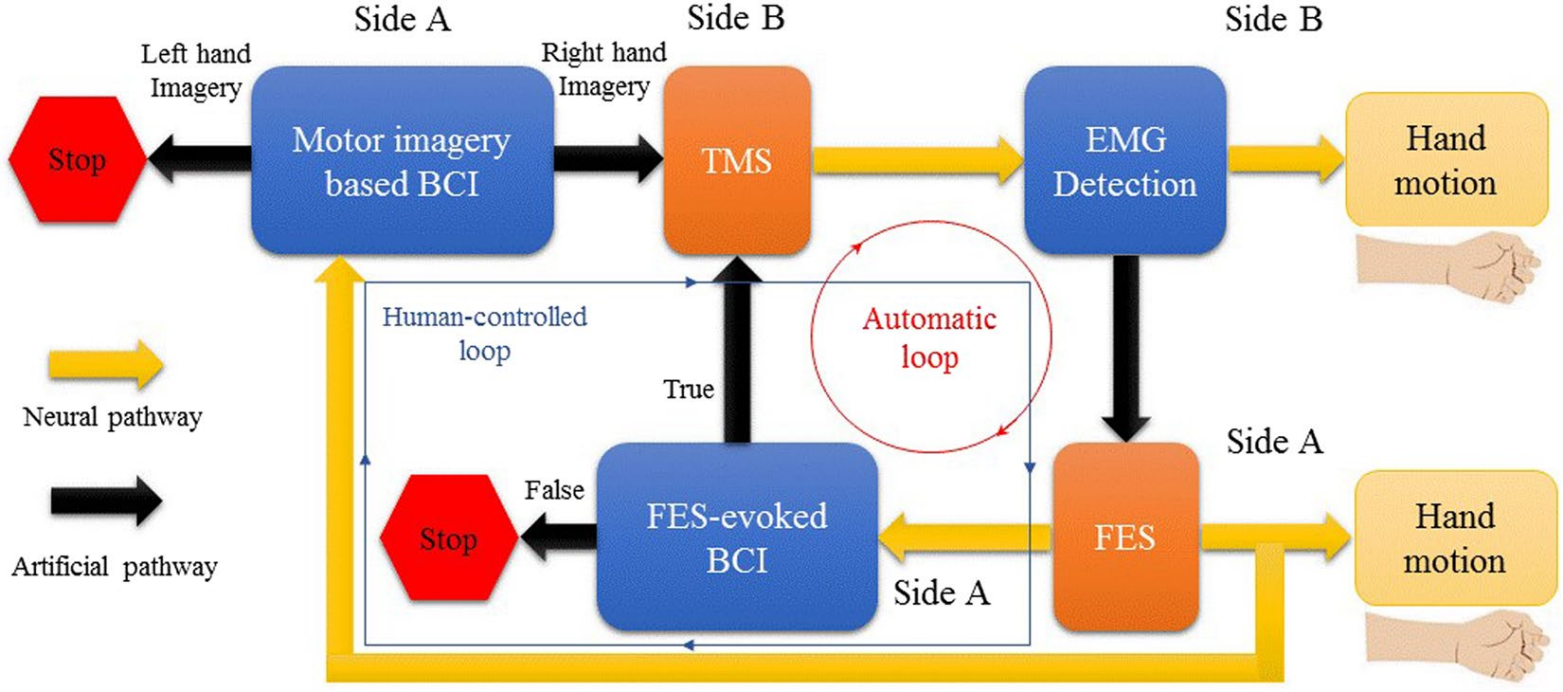
Block diagram of human-controlled loop and automatic control loop. Artificial pathways connect neural pathways of side A and side B as a closed loop.

The participant in side A sat on a comfortable chair in front of a 17-inch display with an EEG cap mounted over sculpt and rest their both hands on a desk (Fig. 6). During each experimental session, two participants had to carry out specific tasks over sequential trials. EEG recording trials comprised of four sub-sections for subjects in side A. At the beginning of the session a blank screen was projected to the subject for 3 s indicating idle state, then a white cross fixation appeared at the center for 1 s altering the subject to get ready for the task which was going to start shortly. Afterward, a red rectangle (approximately 20*10 mm) appeared on the left or right side of the cross for 2.5 s instructing the motor imagery execution. Subjects were instructed to imagine the motion of left or right hand wrist flexion following visual stimulus orientation. Raw EEG data from this time window were instantly sent to the classifier, after 0.5 s the cross vanished and the trial terminated with 1 s of the blank screen, which was vital to remove the possibility of overlapping between varying mental states of consequent trials. Each subject underwent 5 sessions of experiment, 30 trials each (150 trials in total). Raw signals have been clipped in the time window of −1 to 3 second corresponding to the onset of the cue as time zero. Recorded EEG data during first session consisting of 30 trials were used for training. A detailed illustration of the protocol is shown in Fig. 7. Two types of BCIs (independent and dependent BCIs) were used for the subjects in side A. In the human-controlled loop, the independent BCI was motor imagery (MI) based BCI. The subject imagined the right or left hand which controlled the mechanism to start or stop, respectively. The dependent BCI was FES-evoked BCI which was irrelevant to the subject’s voluntary intention and merely used in automatic loop. The MI-based BCI and FES-evoked BCI used the same feature exaction and classification algorithms in this work. Throughout the experimental session, the subject in side B sat on a custom-designed chair in a separated room to barricade any visual or auditory interaction from the subject in side A. Subjects were asked to stay relaxed and wore earplugs to prevent hearing the click sound produced by the TMS coil. TMS-induced MEP can be detected by EMG sensor, which was used as a trigger for FES in side A. In fact, the subject in side B could also dominate the control, i.e. the voluntary wrist flexion and extension could be used as “start” or “stop” commands. For clear and concise representation, the side B-dominant control is not provided in this paper, i.e. the side B was passively involved in the loop.

#### Automatic Loop

The only difference between automatic protocol and human-controlled loop protocol was indeed the BCI part. In other words, independent BCI based on motor imagery served in the human-controlled loop, while dependent BCI based on FES-evoked EEG served in the automatic loop. FES made a muscle contraction and generated passive hand motion that could evoke EEG changes. In this case, BCI could automatically detect and classify the EEG changes. In automatic paradigm, a trial was not finished after FES generated the hand motion. Instead, if classifier’s response for FES-evoked BCI was true, a trigger would be sent to TMS as a trigger and another loop would start as exhibited in Fig. 7. This procedure continued until classification output of FES-evoked BCI, turned out to be false. This is interesting because we could observe the subject in side A had repetitive and continuous right hand movements, just by thinking about moving his hand once at the beginning. However, in the human-controlled paradigm, each trial terminated regardless of the classification result. The difference is obvious as depicted in Fig. 7. The continuity of automatic loop was extremely based upon FES-evoked BCI. In human-controlled loop paradigm, the subject could determine to continue or stop, while In automatic loop, FES would evoke changes of EEG signals and BCI could detect the changes and started next loop automatically, therefore, the action could repeat and never stop if BCI did not provide a wrong detection. The automatic control loop was only tested for pair 6 solely, as a proof of concept.

## Electronic supplementary material

Supplementary Information
