# Supplementary material for: Human-to-human closed-loop control based on brain-to-brain interface and muscle-to-muscle interface
Source: Sci Rep. 2017 Sep 8;7:11001. doi: 10.1038/s41598-017-10957-z (PMC5591235; doi:10.1038/s41598-017-10957-z)
Supplement: Supplementary file 1 — Supplementary Information [file 41598_2017_10957_MOESM1_ESM.pdf]

Title: Human-to-human closed-loop control based on brain-to-brain interface and muscle-to-muscle interface.
Authors: M.Ebrahim M.Mashat, Guangye Li, Dingguo Zhang.

Appendix A

In the current paper, for BCI classification part, after preprocessing (i.e. time segmentation and band-pass filtering of the raw data within the target frequency band), EEG data is treated with well-known CSP and LDA algorithms for feature extraction and feature classification, respectively. Mathematical frameworks of aforementioned algorithms are demonstrated herein. EEG recorded signal is x_n with $ch \times len$ dimensions, which ch shows the number of recording electrodes and len is the number of sampled data points. The aim is to find the most discriminating features for best classification result. **A)** here, CSP filter is actually a filter which use generalized eigenvalue to discriminate two populations of EEG-related signals. Its formulation is:

$$S = \omega^T E \ or \ s(t) = \omega^T e(t) \tag{1}$$

where ω is spatial filter matrix, S is filtered signal matrix, The criterion of CSP is given by:

$$maximize \ tr \ \omega^T \sigma_1 \omega \tag{2}$$
$$subject \ to \ \omega^T (\sigma_1 + \sigma_2)\omega = I$$

where

$$\sigma_{x_n=class1}, \sigma_{x_n=class2} = exp(\frac{x_n X_n^T}{tr x_n X_n^T}), \tag{3}$$

This problem can be solved with generalized Eigenvalue problem. First we decompose,

$$\sigma_1 + \sigma_2 = UDU^T \tag{4}$$

Where U is set of eigen vectors and D is a diagonal matrix of Eigenvalues. After calculations of $P := \sqrt{D^{-1}} U^T$,

$$\hat{\sigma_1} = P\sigma_1 P^T, \hat{\sigma_2} = P\sigma_2 P^T \tag{5}$$

where $\hat{\sigma_1} + \hat{\sigma_2} = I$. Then any orthonormal matrices V satisfy $V^T(\sigma_1 + \sigma_2)V = I$. We can decompose $\hat{\sigma_1} = V\Lambda V^T$, where V is the set of eigen vectors and Λ is diagonal matrix of eigenvalues. A set of CSP filters can be obtained as $\omega = P^T V$, Then:

$$\omega^T \sigma_1 \omega = A = \begin{bmatrix} \gamma_1 & \cdots & 0 \\ \vdots & \ddots & \vdots \\ 0 & \cdots & \gamma_{ch} \end{bmatrix} \tag{6}$$

Where $\gamma_1 \geq \gamma_2 \geq \cdots \gamma_{ch}$. Therefore, first CSP filter provides maximum variance of class 1 and last CSP filter provides maximum variance of class 2. Filtered signal matrix is given by:

$$s(t) = \omega_{csp}^T e(t) = (s_1(t), \cdots, s_d(t))^T \tag{7}$$

Then feature vector x calculated by:

$$x_i = log(\frac{[var s_i(t)]}{\Sigma_1^{\sigma}(var s_i(t))}) \tag{8}$$

B) LDA is a recognized binary classification method. It is based on mean vectors and covariance matrices of patterns for individual classes. If a transformation of a d-dimensional vector x to scalar z is considered: $z = \omega^T x$, LDA would give an optimal projection of w so that the distribution of z is easily discriminated. The criterion of LDA is given by:

$$maximize \ J(\omega) = \frac{(m_1 - m_2)^2}{s_1 + s_2} \tag{9}$$

where m_1, m_2 denotes averages for $z_n \in [class1] or [class2]$, and s_1, s_2 denotes the variances of $z_n \in [class1] or [class2]$, respectively. Afterwards, $S_B = (\mu_1 - \mu_2)[(\mu_1 - \mu_2)]^T$, $S_w = \sigma_1 + \sigma_2$, where μ is the mean vector of $x_n \in [class1] or [class2]$ and σ is covariance matrix of $x_n \in [class1] or [class2]$, the cost function will be $J(\omega) = \frac{\omega^T S_B \omega}{\omega^T S_w \omega}$.

$$\omega^T x \geq z_0 = x \in [Class1], \tag{10}$$

$$\omega^T x < z_0 = x \in [Class2], \tag{11}$$

for instance, $z_0 = (m_1 + m_2)/2$ could be usable.
